# Congenital Sublingual Dermoid Cyst Mimicking a Recurrent Ranula in a Pediatric Patient: A Case Report

**DOI:** 10.7759/cureus.76570

**Published:** 2024-12-29

**Authors:** Omar I Alanazi, Mohammed S Halawani, Sara E Marhoon, Ali H Ali, Baraa Awad

**Affiliations:** 1 College of Medicine, King Saud Bin Abdulaziz University for Health Sciences, Riyadh, SAU; 2 Division of Otolaryngology - Head and Neck Surgery, King Abdullah Specialized Children's Hospital, King Abdulaziz Medical City, National Guard Health Affairs, Riyadh, SAU; 3 Department of Pediatrics, Mansoura University Hospital, Mansoura, EGY; 4 Department of Pediatrics, Qatif Central Hospital, Eastern Health Cluster, Qatif, SAU; 5 Department of Otolaryngology - Head and Neck Surgery, College of Medicine, King Saud Bin Abdulaziz University for Health Sciences, King Abdullah International Medical Research Center, Ministry of National Guard Health Affairs, Jeddah, SAU

**Keywords:** case report, dermoid cyst, floor of mouth, ranula, sublingual swelling

## Abstract

In pediatrics, sublingual lesions are not a common disease. Due to the similarity in their clinical and radiological features, they present a diagnostic challenge. Despite the advancement in imaging techniques, the accurate preoperative diagnosis of sublingual lesions may fail. In this study, we present a case of a 13-year-old male referred to our surgical department with a recurrent painless mass in the floor of the mouth, which had been previously diagnosed as a ranula and treated at another medical facility. However, the patient experienced a recurrence six months later. Upon examination, a firm left sublingual mass extending into the neck was detected. Magnetic resonance imaging was performed, which was suggestive of a ranula. The lesion was surgically excised transorally, and histopathological examination unexpectedly revealed the diagnosis of a dermoid cyst. This case highlights the importance of considering dermoid cysts when a sublingual lesion is encountered, emphasizing the need for histopathology to confirm the diagnosis.

## Introduction

Although lesions of the pediatric floor of the mouth are relatively uncommon, these lesions have a wide range of differential diagnoses. They include dermoid cysts, foregut duplication cysts, ranulas, epidermoid cysts, and heterotopic gastrointestinal cysts, in decreasing order of frequency, according to a recent systematic review [1]. Although the dermoid cyst is the most common among them, it is still considered rare, accounting for only 0.01% of all oral cysts [2]. Due to their common anatomic location, these lesions often present similarly, with symptoms such as tongue elevation and fullness. As the condition progresses, it can lead to difficulties with breathing, swallowing, and speech [3]. To narrow down these differential diagnoses, imaging techniques such as ultrasound (US), magnetic resonance imaging (MRI), and computed tomography (CT) are utilized. These modalities help characterize the lesion and provide a comprehensive assessment of deep structure involvement, thereby facilitating management decisions [4]. However, sublingual dermoid cysts are often indistinguishable from ranulas based on imaging alone due to their several similarities. This can result in ineffective treatment and a higher risk of recurrence [5].

In this context, we present a case of a congenital sublingual dermoid cyst that mimicked a recurrent ranula. This case underscores the challenges in achieving an accurate diagnosis and emphasizes the importance of employing diagnostic tools to minimize misdiagnosis and prevent mistreatment.

## Case presentation

**Figure 1:**
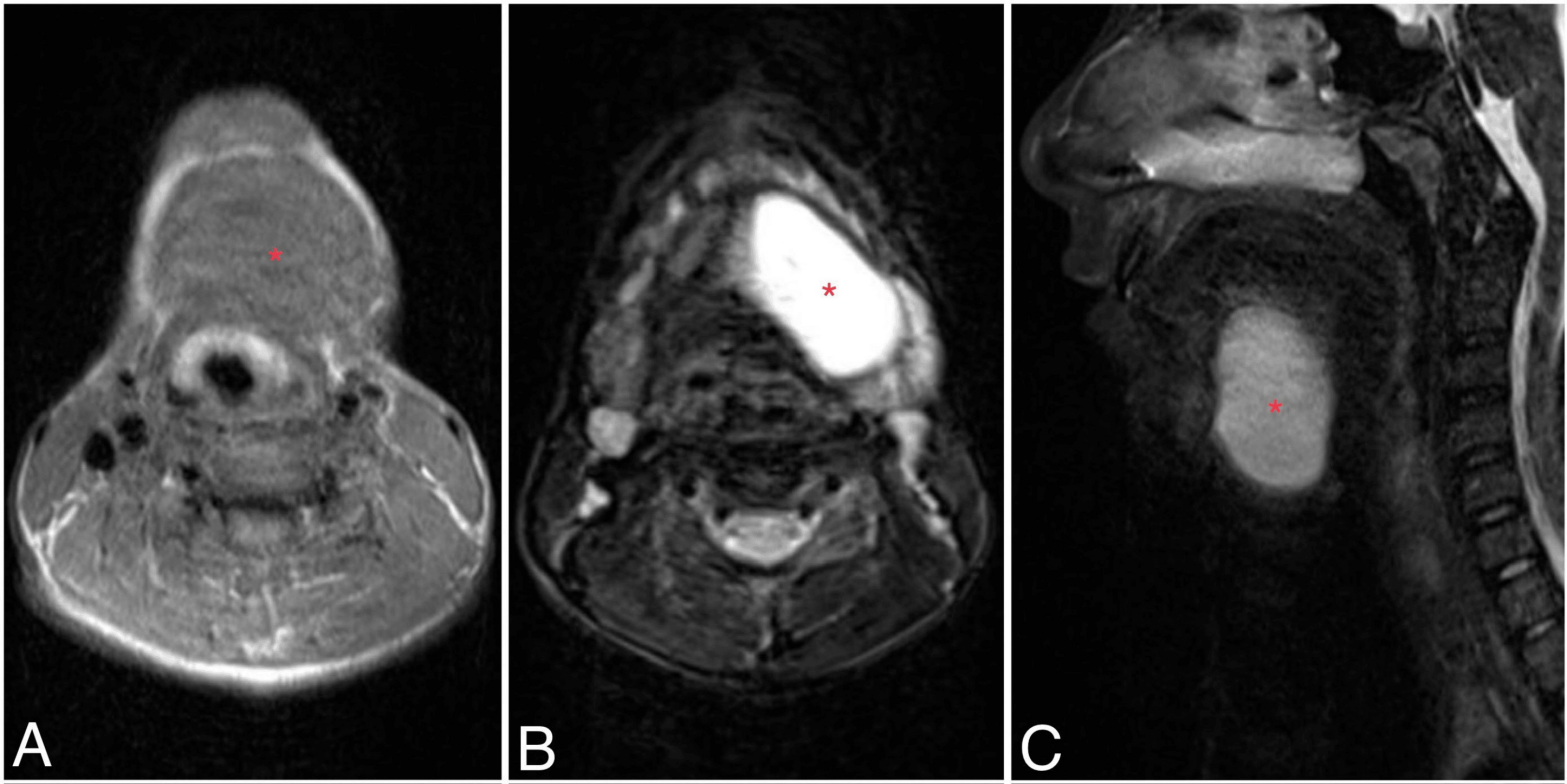
MRI demonstrates a well-defined, thin-walled cystic mass arising from sub-mental space on the left side and extending to the midline measuring 2.5 x 5 x 4.5 in axial, anterior-posterior, and craniocaudal dimensions, respectively. (A) The lesion is characterized by having a homogenous iso-intense signal in the T1-weighted image. (B, C) The lesion is non-enhanced with contrast, and hyper-intense in the T2-weighted image.

A 13-year-old male was referred to our surgical department from another center with a painless floor-of-the-mouth mass, which was diagnosed as a ranula and treated at another medical facility. However, the mass recurred within six months. Otherwise, the patient’s medical history was unremarkable. A firm left sublingual mass extending into the neck was identified upon examination. No abnormalities were identified in the laboratory workup. A neck MRI demonstrated a well-circumscribed, thin-walled cystic lesion without solid components arising from the left sublingual space (Figure 1). The lesion measured 2.5 x 5 x 4.5 cm in axial, anteroposterior, and craniocaudal dimensions, respectively. These findings were suggestive of a ranula.

**Figure 2:**
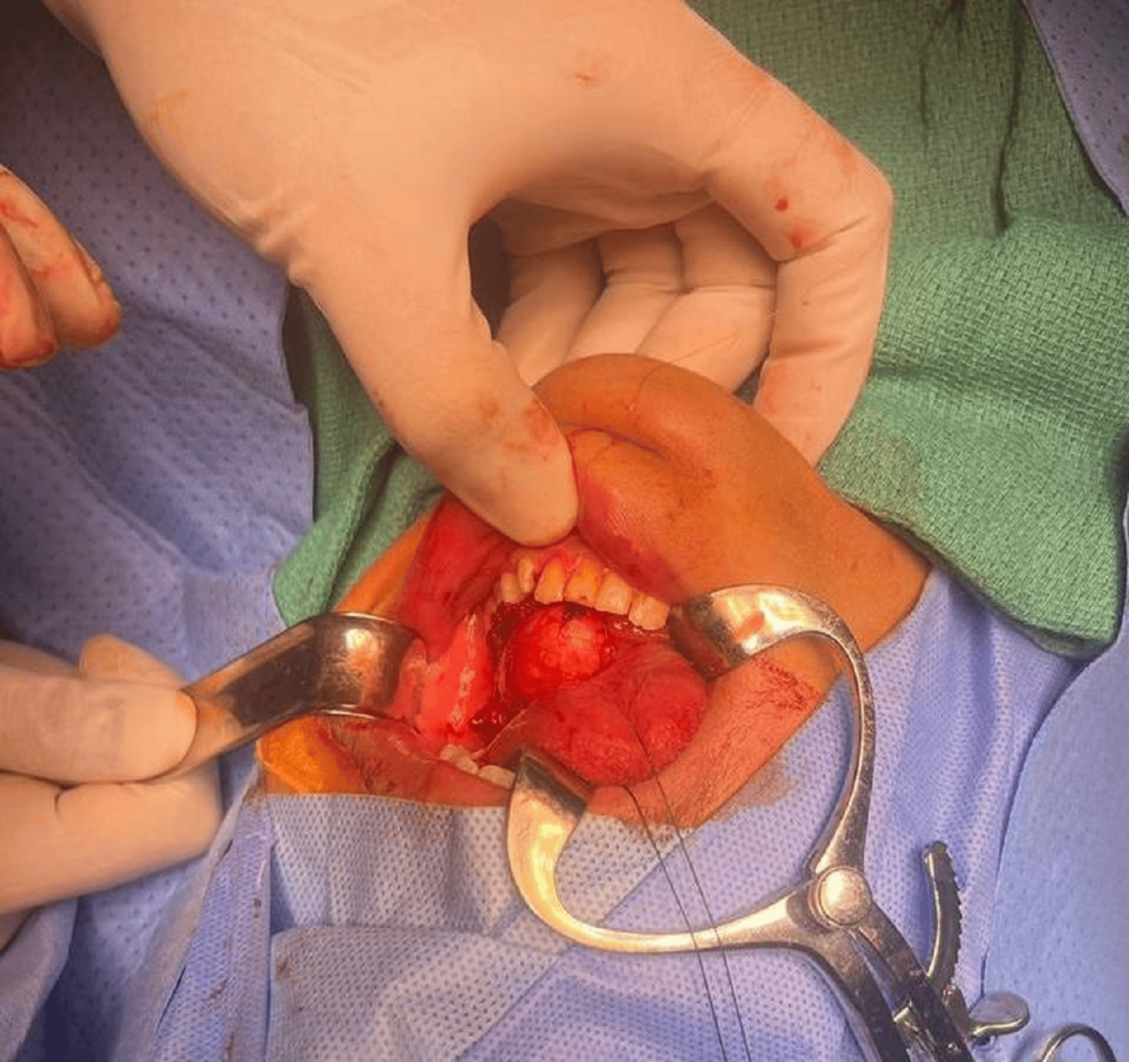
Swelling on the left side of the floor of the mouth.

**Figure 3:**
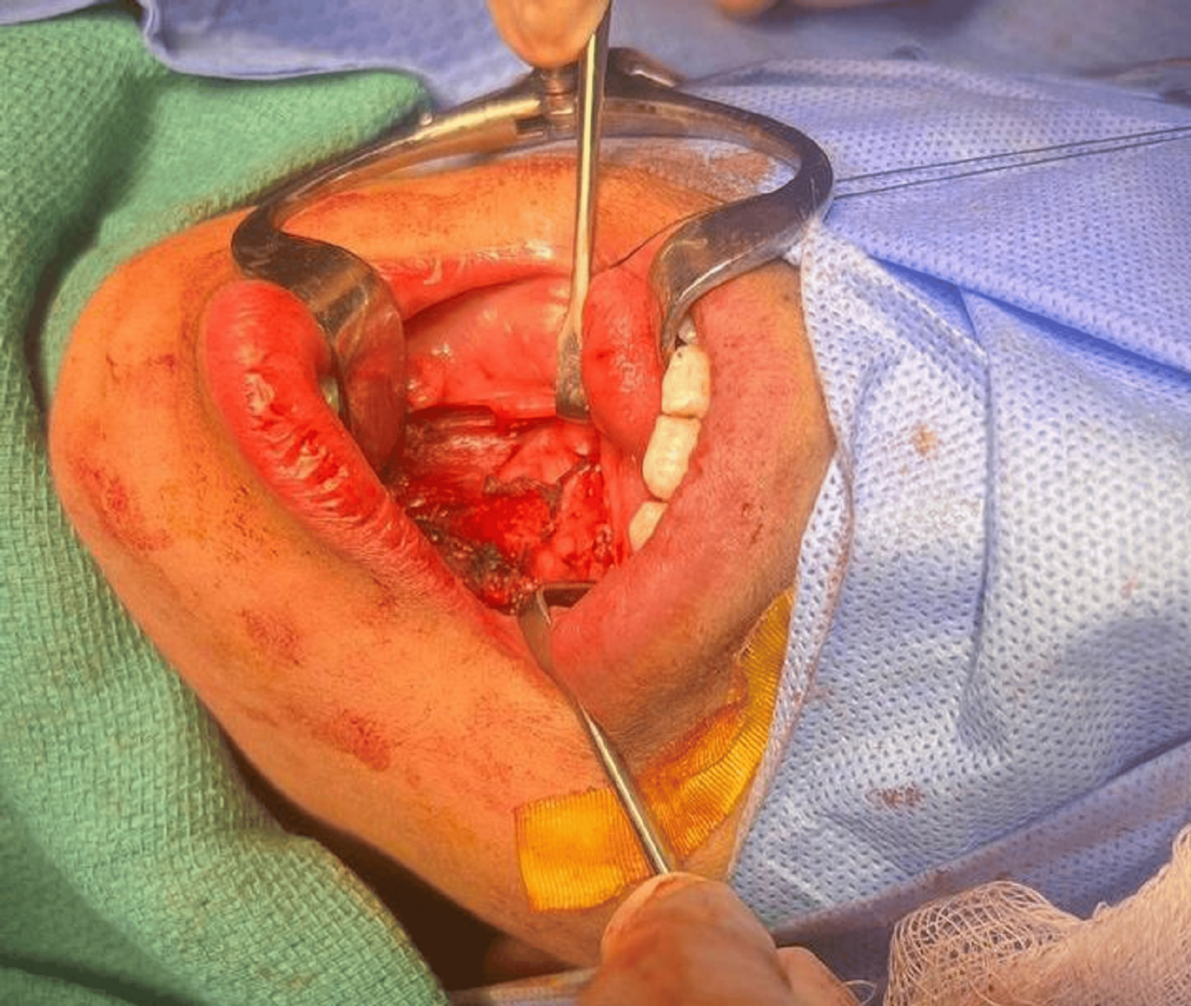
Post-transoral excision of the mass.

The patient underwent surgical excision of the ranula under general anesthesia with nasal intubation. Muscle relaxants were avoided. The patient was prepped and draped in the usual sterile manner, exposing the mouth and neck area. Upon dental retraction, a swelling was observed on the left floor of the mouth (Figure 2). A 3-cm mucosal incision was made on the bulging area of the mass. Dissection was performed around the mass until its wall was identified. The dissection continued until the lingual nerve, which was preserved and kept intact with the assistance of submental palpation to apply pressure to the mass, was reached. The mass gradually emerged from the floor of the mouth. The dissection continued anteriorly, posteriorly, to the right, and to the left until the mass was fully excised (Figure 3). Another examination demonstrated normal vascular and nerve structures. There was no injury to the submandibular duct, nerve, or vessels.

**Figure 4:**
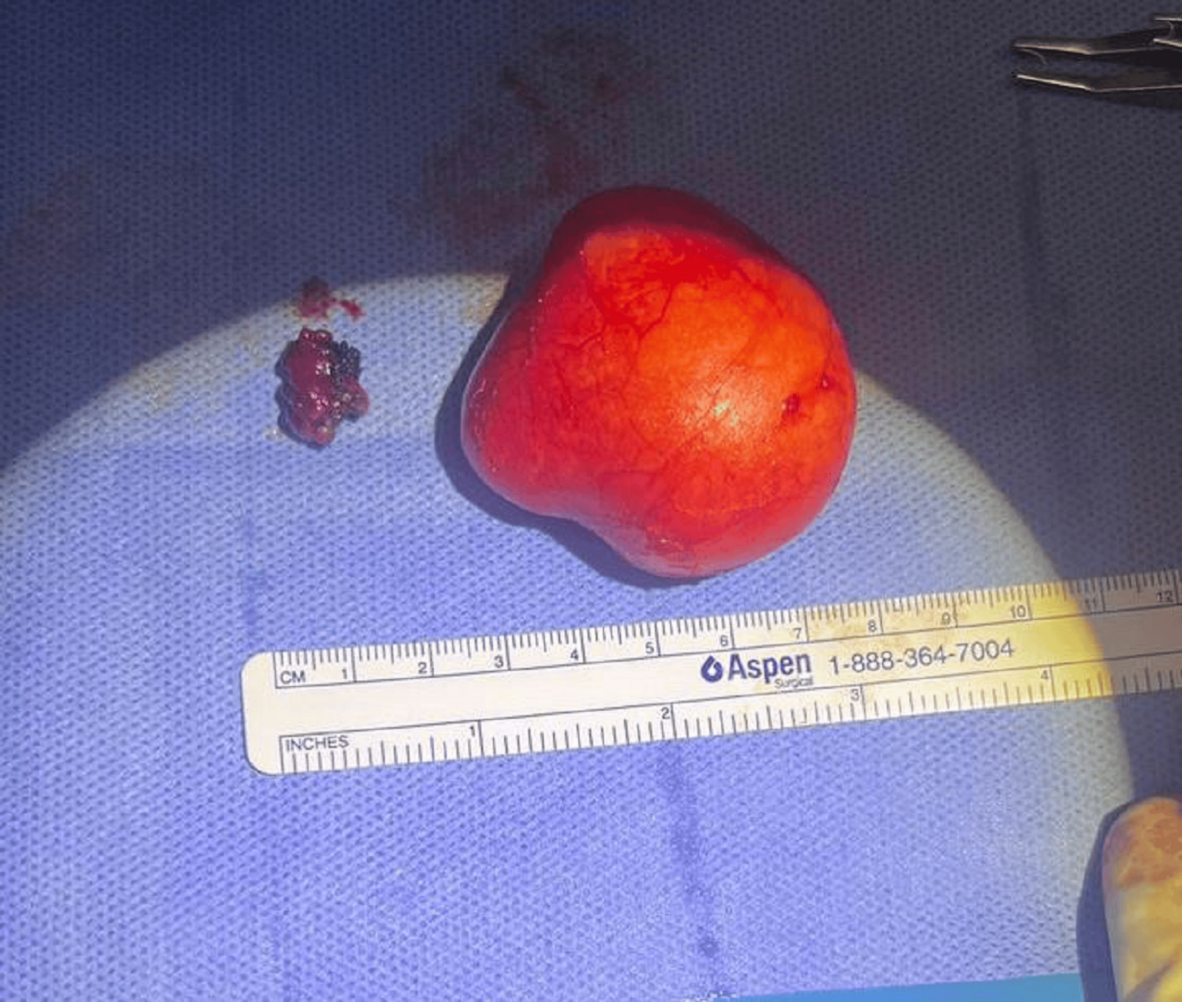
Postoperative specimen.

**Figure 5:**
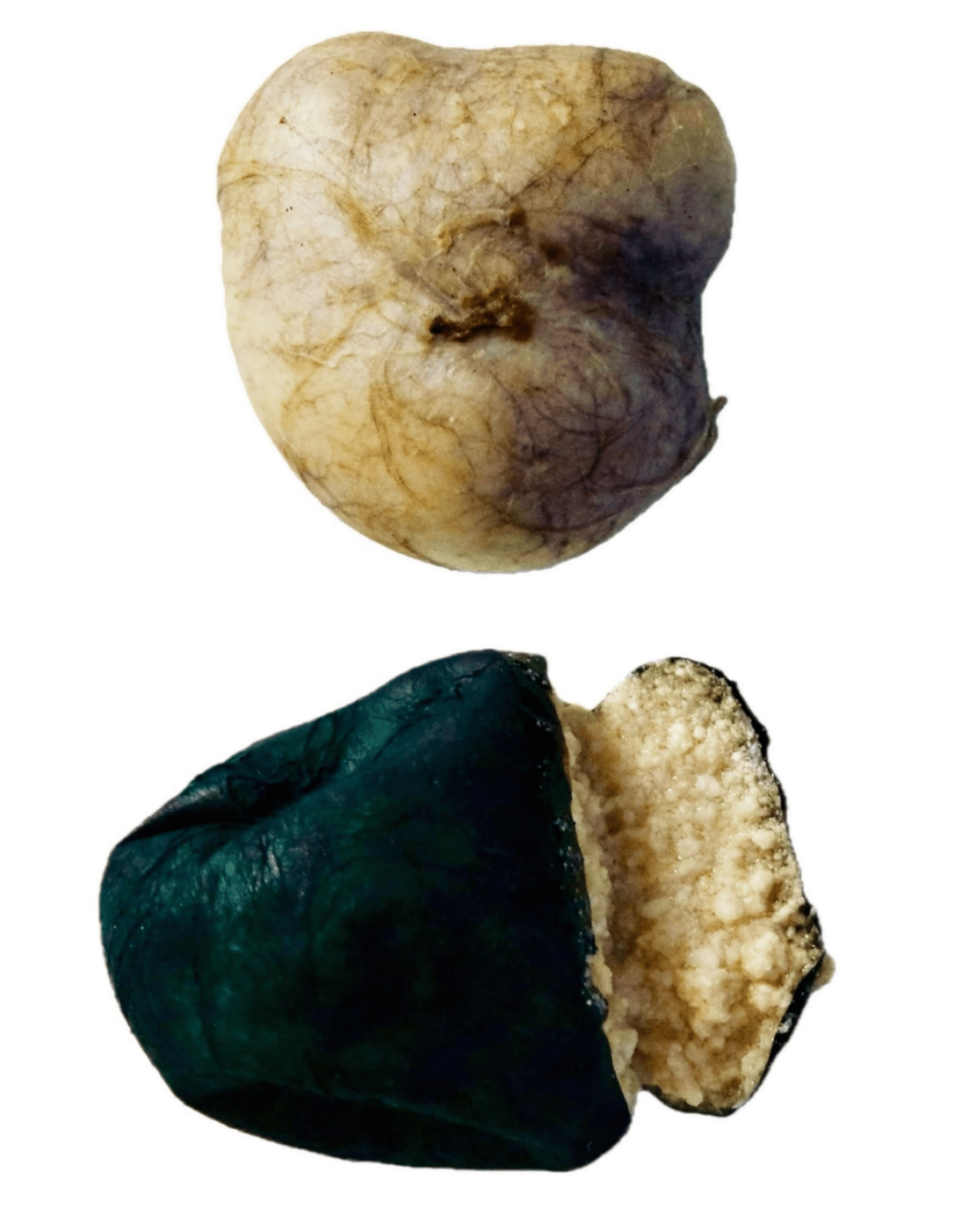
Gross (macroscopic) picture: An intact cyst measuring 5.0 x 4.5 x 2.2 cm and weighing 38 grams. The outer surface is gray-tan and smooth (inked green). The cut surface shows an unilocular cyst filled with white-tan greasy materials.

**Figure 6:**
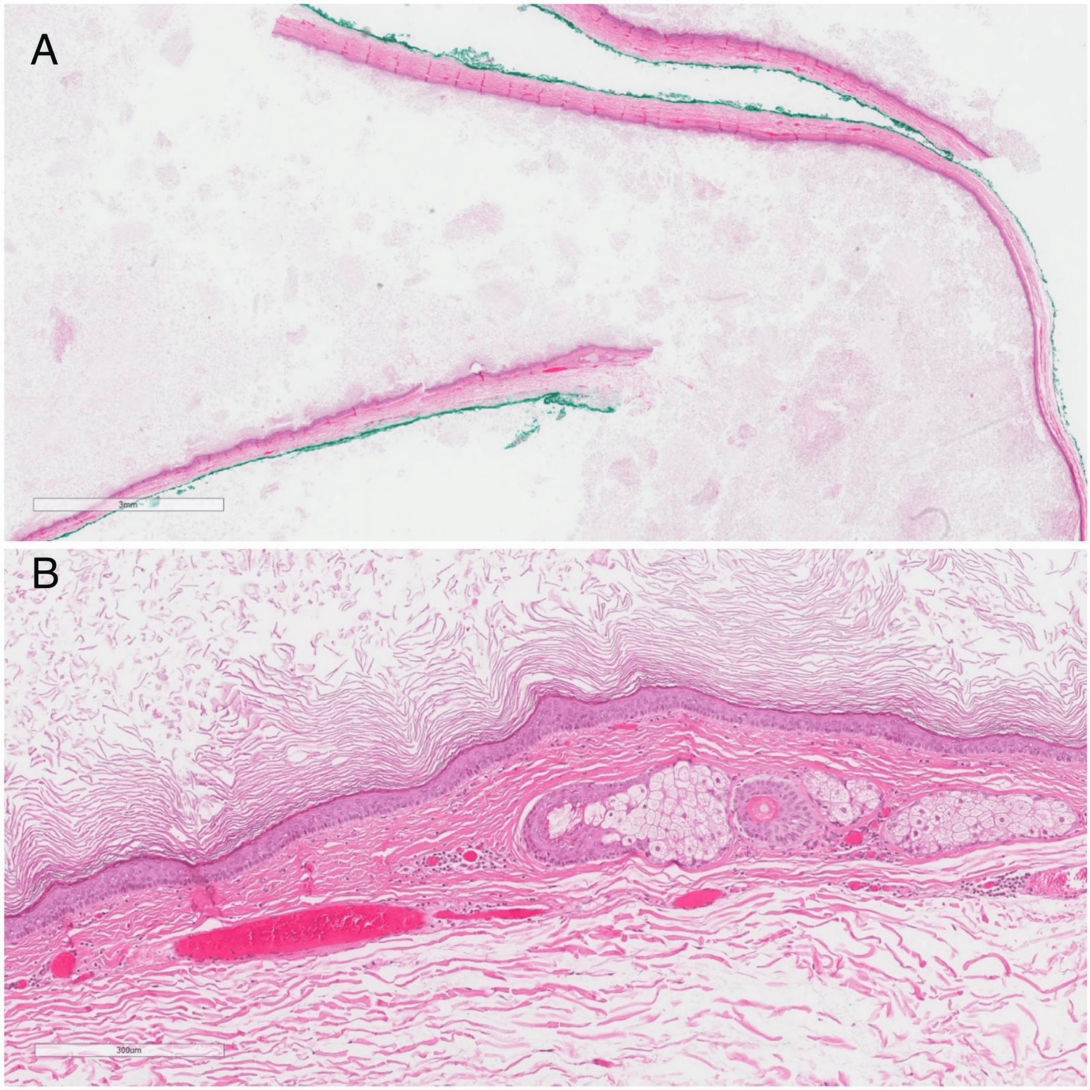
Microscopic picture: Histological section showing a cyst wall lined by keratinizing stratified squamous epithelium with skin adnexal structures, sebaceous glands and hair follicles (pilosebaceous structures), and a lumen filled with keratin (hematoxylin and eosin). (A) Magnification x10. (B) Magnification x100.

Hemostasis was achieved by packing and using bipolar cautery. Dissection was performed using a fine-tip mosquito and Colorado-tipped monopolar cautery. The sublingual gland was identified and removed on the left side to decrease the chance of recurrence. The mucosal layer at the floor of the mouth was closed with 3-0 Vicryl sutures. Irrigation was performed, and observation for five minutes revealed no bleeding. The patient was extubated without complications and transferred to the general ward in stable condition. Figure 4 demonstrates the postoperative specimen that was submitted for histopathological examination. Unexpectedly, the histopathological examination revealed a dermoid cyst rather than a ranula (Figures 5, 6). The cyst’s outer surface was tan-gray, smooth, and congested. Serial sectioning revealed a unilocular cystic lesion with a thin wall, filled with tan-white materials. No pathological changes were noted in the excised minor salivary gland.

Two days after the operation, the patient was discharged with a prescription for ibuprofen, acetaminophen, and clindamycin. Follow-up appointments revealed a stable condition.

## Discussion

Despite their rarity, sublingual lesions in the pediatric age group present a diagnostic challenge. One of the obstacles that physicians encounter is that sublingual dermoid cysts are commonly misdiagnosed as ranulas [6]. In a 20-year retrospective study on pediatric sublingual epidermoid and dermoid cysts, it was found that preoperative diagnoses are often inconclusive. Approximately 33% of cases were categorized as nondiagnostic “cystic masses,” 25% were misdiagnosed as ranulas, which is the most common misdiagnosis, 17% were thought to be lymphatic malformations, and only 17% were accurately diagnosed preoperatively [6]. In our case, the cyst was preoperatively diagnosed as a recurrent ranula, but the pathology report later confirmed the lesion to be a dermoid cyst.

A congenital dermoid cyst forms due to the entrapment of ectoderm and mesoderm along embryonic fusion planes [7]. For example, a sublingual dermoid cyst develops during the fusion of the first and second branchial arches [8]. These cysts contain heterogeneous material, including mature skin appendages, stratified squamous epithelium, and, possibly, keratin and hair [7]. Dermoid cysts account for only 0.01% of all oral cysts and 1.6% of all dermoid cysts [2]. Dermoid cysts of the floor of the mouth are commonly diagnosed between the first and third decades of patients’ lives [8]. Our patient was 13 years old, which supports this.

On the other hand, a ranula is a pseudocyst that develops on the floor of the mouth, typically due to trauma. This trauma causes the rupture of the excretory salivary duct, leading to the extravasation and accumulation of saliva within the tissue. Congenital ranulas occur relatively scarcely, with an occurrence rate of approximately 0.74% [9]. The distinguishing features between a dermoid cyst and a ranula are summarized in Table 1.

**Table 1:** Summary of key distinguishing features of dermoid cyst and ranula. Source: Martins et al. [10].

	Common features	Dermoid cyst features	Ranula features
Clinical presentation	Painless, soft, and compressible lesions	May present a yellow hue seen through the skin, and is typically located on the midline	Painless, soft, and compressible lesions; blue coloration, but deeper lesions may be normal in color, and usually located lateral to the midline
CT	Thin-walled, hypodense, unilocular mass	With pathognomonic "sack of marbles" appearance caused by multiple hypo-attenuating fat nodules	Without areas of fat attenuation
MRI	High intensity on T2-weighted images	T1-weighted imaging demonstrates a variable signal depending on fat content, and high intensity on T2-weighted images	Plunging ranulas often exhibit a slight extension of the lesion into the sublingual space, known as a "tail sign"
Histopathology aspects	-	Encapsulated lesion with soft, yellow material, often with cutaneous elements such as hair in the interior	Collected mucin, lacking epithelial lining (mucocele)
Treatment	-	Complete enucleation	Removal of the feeding sublingual gland and/or marsupialization

Due to the infrequent occurrence of these lesions, clear guidelines for accurate preoperative imaging have yet to be established. Nevertheless, imaging techniques such as US, MRI, and CT are valuable for assessing the lesion’s location, extent, relationship with surrounding structures, and potential malignancy, with MRI being superior to other imaging modalities [1]. A five-year retrospective study on floor-of-mouth masses in children indicated that, while MRI is the preferred imaging modality, its accuracy in diagnosing dermoid cysts is comparable to clinical diagnosis. This is due to the unpredictable content of dermoid cysts [3]. In our case, despite using MRI - the preferred imaging method for sublingual lesions - the result was inaccurate, leading to a preoperative misdiagnosis.

Complete surgical excision under general anesthesia is strongly recommended for treating young patients with sublingual dermoid cysts, even if the lesion is small, to avoid trauma and potential behavioral impacts [11]. Surgical excision can be performed either transorally, which is typically used for masses located above the mylohyoid muscle, or transcervically for masses situated more submentally. Although the transoral approach offers better aesthetic outcomes, the transcervical approach is preferred in cases of cyst infection, difficult airway access, or poor visibility during transoral surgery [1]. Uniquely in our case, the excision was performed entirely transorally without any injury to the submandibular duct, nerve, or vessels. Additionally, the sublingual gland was removed to minimize the risk of recurrence.

The prognosis for dermoid cysts is generally excellent, with negligible rates of recurrence, postoperative infection, and malignant transformation. Recurrence primarily occurs due to residual cyst remnants, especially in cases where marsupialization is used, which is more favorable for ranulas, as dermoid cysts have true epithelial linings that may lead to incomplete removal [12]. Our patient initially underwent incision and drainage of the lesion at another medical facility, which led to a recurrence, an event considered rare.

## Conclusions

This case report highlights the challenges that may be encountered in the diagnosis of sublingual dermoid cysts, such as their potential to mimic ranulas. Despite advancements in imaging modalities, preoperative diagnosis often remains inconclusive, as in our case, which leads to a misdiagnosis. This is attributed to the similarities of sublingual lesions regarding their clinical and radiological features. All of these factors emphasize the importance of surgical excision and histopathological examination in reaching a definitive diagnosis and avoiding future recurrence. Future studies are needed to establish a more definitive preoperative diagnostic algorithm for achieving proper management and avoiding the dilemma of recurrence.
